# Reduced discomfort during high-definition transcutaneous stimulation using 6% benzocaine

**DOI:** 10.3389/fneng.2014.00028

**Published:** 2014-07-11

**Authors:** Berkan Guleyupoglu, Nicole Febles, Preet Minhas, Christoph Hahn, Marom Bikson

**Affiliations:** ^1^Department of Biomedical Engineering, Neural Engineering Laboratory, The City College of the City University of New YorkNew York, NY, USA; ^2^Biomedical Engineering, University of Applied Sciences HamburgHamburg, Germany

**Keywords:** transcutaneous, tDCS, HD-tDCS, sensation, direct current stimulation

## Abstract

**Background:** High-Definition transcranial Direct Current Stimulation (HD-tDCS) allows for non-invasive neuromodulation using an array of compact (approximately 1 cm^2^ contact area) “High-Definition” (HD) electrodes, as compared to conventional tDCS (which uses two large pads that are approximately 35 cm^2^). In a previous transcutaneous study, we developed and validated designs for HD electrodes that reduce discomfort over >20 min session with 2 mA electrode current.

**Objective:** The purpose of this study was to investigate the use of a chemical pretreatment with 6% benzocaine (topical numbing agent) to further reduce subjective discomfort during transcutaneous stimulation and to allow for better sham controlled studies.

**Methods:** Pre-treatment with 6% benzocaine was compared with control (no pretreatment) for 22 min 2 mA of stimulation, with either CCNY-4 or Lectron II electroconductive gel, for both cathodal and anodal transcutaneous (forearm) stimulation (eight different combinations).

**Results:** Results show that for all conditions and polarities tested, stimulation with HD electrodes is safe and well tolerated and that pretreatment further reduced subjective discomfort.

**Conclusion:** Pretreatment with a mild analgesic reduces discomfort during HD-tDCS.

## Introduction

Transcranial Direct Current Stimulation (tDCS) is an investigational neuromodulation technique that uses scalp electrodes to deliver 1–2 mA of direct current for 10–20 min (Guleyupoglu et al., 2013; Villamar et al., 2013). Whereas conventional tDCS uses two large pads (approximately 35 cm^2^ contact area, although not limited to this size), High-Definition tDCS (HD-tDCS) uses an array of smaller HD electrodes (approximately 1 cm^2^ contact area) to deliver current in a more target specific manner (Datta et al., 2008, 2009; Dmochowski et al., 2011; Guleyupoglu et al., 2013). Previously we showed that control over HD electrode parameters, namely shape, electrode material, and gel, allows for tolerated stimulation with no lasting skin irritation (Minhas et al., 2010). Several clinical studies have supported the safety and tolerability of HD-tDCS (Dundas et al., 2007; Nitsche et al., 2009; Borckardt et al., 2012; Caparelli-Daquer et al., 2012; Kuo et al., 2013); in some cases pre-treatment with mild topical anesthetic containing a low-concentration of benzocaine was used. Here we directly quantified if pre-treatment with a cream containing 6% benzocaine was effective in reducing discomfort across the forearm during 22 min of stimulation at 2 mA. The forearm was used during the investigation as it provides a model that does not involve unintended direct stimulation of the brain. Though clinical trials support HD-tDCS as a well-tolerated technique, approaches to reduce discomfort and tingling during stimulation may further enhance tolerability and sham reliability.

## Methods

Ten healthy subjects, both male (5) and female (5) with ages ranging from 18 to 35, participated in this study. Each subject gave informed consent before being included in this study. This study was approved by the Institutional Review Board of The City College of New York. A total of eight different conditions were tested in this series using combinations of anodal/cathodal stimulation polarity, CCNY-4/Lectron electroconductive gel, and control/pretreatment conditions (e.g., anodal stimulation with CCNY-4 gel and no pretreatment). Each subject underwent stimulation under all eight conditions in random order and was blinded to the set-up in regards to anodal/cathodal and which electrogel was used. Stimulation was administered using a constant current stimulator (Schneider, Germany or Soterix Medical Inc. 1 × 1 tDCS, New York, NY); 2 mA of current was delivered to the forearm for 22 min (20 min stimulation, 2 min post-stimulation), with a 10 s ramp up before stimulation, and a 10 s ramp down before completion of the stimulation. Ag/AgCl sintered ring electrodes were used (one active and two returns); the active electrode was positioned in the center of the forearm, and the two returns electrodes were positioned on the upper and lower forearm at approximately 5 cm apart, immersed in a high volume of gel—the use of two electrodes with high contact area resulted in sensation being restricted largely to the center active electrode; thus stimulation polarity, “anodal” or “cathodal” is used here in references to the center electrode polarity. The electrode-skin contact area was either pretreated with 0.2 ml of 6% benzocaine (Lanacane brand) (and left on the skin before applying gel and stimulation for approximately 8 min) or was left untreated (control). The sintered Ag-Ag/Cl electrodes (550025, Ring Electrode Stens Corp.) were held in place using electrode holders (Soterix Medical, New York, NY, HD-1) and were immersed in either CCNY-4 (custom made) or Lectron (Lectron II, Pharmaceutical Innovation Inc., Newark, NJ, USA). Subjects were asked to rate the discomfort felt, using a pain scale from 0 to 10 (0 being no pain and 10 being the worst imaginable pain ever), after stimulation ramped up, every minute during stimulation and after stimulation ramped down. Subjects were also asked to describe the sensation that they were feeling (“burning”, “prickling”, etc.). Subjects were alerted when stimulation was initiated but not when stimulation terminated. After stimulation was terminated, a period of 1 week was used as a “wash-out” period prior to stimulating the subject again. To analyze the three independant factors (gel type, polarity, and whether or not pretreatment was applied) in this experiment, a 3-way repeated measures ANOVA was utilized.

## Results

**Figure 1:**
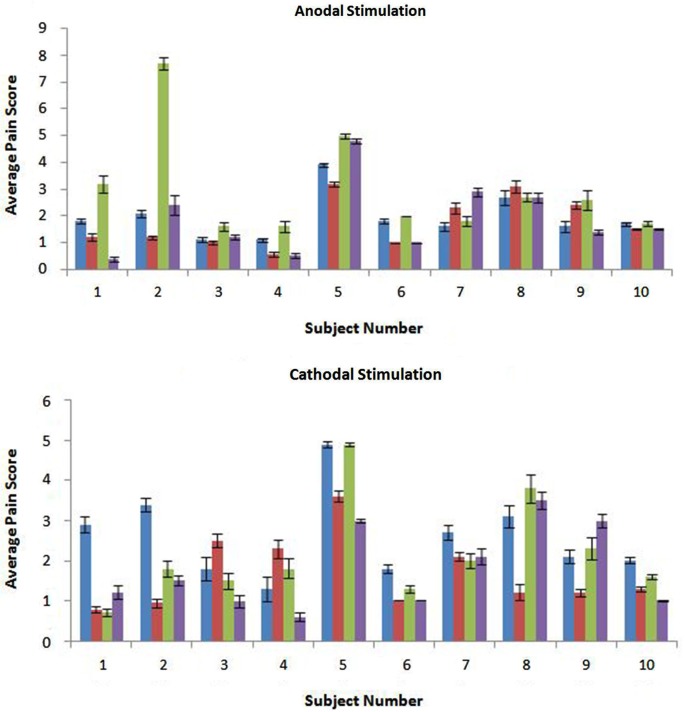
**The anodal and cathodal scores are shown with the average for each subject and error bars are representative of standard error**. From left to right the conditions shown are: CCNY-4/No Pretreatment (in blue), CCNY-4/Pretreatment (in red), Lectron/No Pretreatment (in green), and Lectron/Pretreatment (in purple). There was a significant difference between the control and pretreatment groups (*p* = 0.0157).

Stimulation was well tolerated across subjects and conditions (Figure 1). Regardless of polarity and gel type, pretreatment reduced subject sensitivity to stimulation (*p* = 0.02,* F* = 6.12). Further investigation into the effects of polarity (*p* = 0.80, *F* = 0.06) and gel type (*p* = 0.45, *F* = 0.58) show no significant difference on subject sensitivity to stimulation. The interaction effect between these three factors was also investigated. The interaction between polarity and gel type did not significantly reduce subject sensitivity to stimulation (*p* = 0.16, *F* = 1.97) as well as the interaction between polarity and pretreatment (*p* = 0.96, *F* < 0.01). The interaction between gel type and pretreatment was also not significant in the reduction of sensitivity (*p* = 0.73, *F* = 0.12). Finally, the interaction between the three factors was not significant in the reduction of subject sensitivity to stimulation (*p* = 0.19, *F* = 1.74).

## Discussion

The motivation behind this study was to reduce the level of discomfort that the subjects feel during stimulation. In this study, pretreatment with 6% benzocaine was shown to decrease discomfort for subjects during stimulation. Benzocaine was chosen for this investigation since it is a mild local anesthetic that is typically used as a pain reliever which could help reduce any discomfort that subjects may have during stimulation. The most common sensations felt by subjects were itching, tingling and prickling however, these sensations were not strictly correlated with the pain score reported. To assess the role of carrier and also electrode design, we evaluated the effect of aloe and hydrocortisone on discomfort. In the experiments we combined anode/cathode and considered two electrode designs (Tin and Ag/AgCl). In the experiments (*n* = 8) we observed no effect on discomfort by carrier for either electrode design (*p* = 0.18, *p* = 0.25). Since there was no observed effect on discomfort by carrier, the control for this design with no pretreatment was used. However, it is still possible that there is a placebo effect present.

Current flow distribution and electrochemical changes are critically dependant on all aspects of electrode design (Dundas et al., 2007; Minhas et al., 2010; Kronberg and Bikson, 2012; Caytak et al., 2013). It is important to emphasize that the results of this study are specific to our validated HD electrode design (Minhas et al., 2010; Borckardt et al., 2012), and further more apply only to the dose tested (e.g., up to 22 min, 2 mA, one session), such that, as with any treatment, adoption of new electrode designs, increased stimulation intensity, or repetition warrants explicit testing. A recent report showed no effect on sensation using lidocaine, an agent similar to benzocaine, when testing conventional tDCS electrodes—indicating the efficacy of anesthetic is dependent on electrode design or montage (Guarienti et al., 2014).

## Conflict of interest statement

Dr. Marom Bikson is the inventor of technologies dealing with transcranial electrical stimulation for which The City College of New York holds patents.
